# Should miliary tuberculosis be considered as a possible cause of infertility in the new era: a case report and literature review

**DOI:** 10.3389/fmed.2025.1520644

**Published:** 2025-01-30

**Authors:** Aleksandra Cvetkovic, Ana Blanka Protic, Jelena Jovanovic, Tatjana Adzic Vukicevic

**Affiliations:** ^1^Clinic of Pulmonology, University Clinical Center of Serbia, Belgrade, Serbia; ^2^Faculty of Medicine, University of Belgrade, Belgrade, Serbia

**Keywords:** miliary tuberculosis, pregnancy, *in vitro* fertilization, latent tuberculosis, low TB burden country

## Abstract

**Introduction:**

Miliary tuberculosis (MTB) is a potentially lethal form of tuberculosis that can occur in pregnant women, especially those who have conceived by *in vitro* fertilization (IVF).

**Case description:**

A 28-year-old, female patient, after IVF’s fourth attempt, at the end of the first trimester, developed a dry cough, high fever, abdominal pain, and vaginal bleeding, which led to the pregnancy termination without resolution of systemic symptoms despite various antibiotics. Because of the appearance of headaches, brain nuclear magnetic resonance (NMR) was done, and diffuse nodular brain lesions were found, which were initially interpreted as metastatic cancer disease. Afterward, the miliary changes were discovered in various organ systems, and the presence of *Mycobacterium tuberculosis* was confirmed. The antituberculosis treatment was initiated with the standard antituberculosis regimen with excellent clinical response and resolution of miliary changes.

**Conclusion:**

Miliary tuberculosis is more common in cases of pregnancies related to IVF. It should be taken into consideration as a possible risk for infertility in the presence of nonspecific symptoms. Screening methods for latent tuberculosis in IVF patients are needed even in a low-burden TB country.

## Introduction

Miliary tuberculosis (MTB) represents a form of tuberculosis that originates from the hematogenous spread of *Mycobacterium tuberculosis*. According to literature data (1), it is usually found in adults and is due to recent infection or reactivation of latent tuberculosis. The predominant symptoms are often nonspecific and dependent on the most affected organs. If it is not recognized and treated accordingly, miliary tuberculosis could be fatal. Considering that more than 200.000 active TB cases were registered among pregnant women worldwide, along with the increased use of *in vitro* fertilization (IVF) methods and the increased number of MTB cases, it becomes obvious that this is an important topic for further investigation (2).

## Case description

**FIGURE 1:**
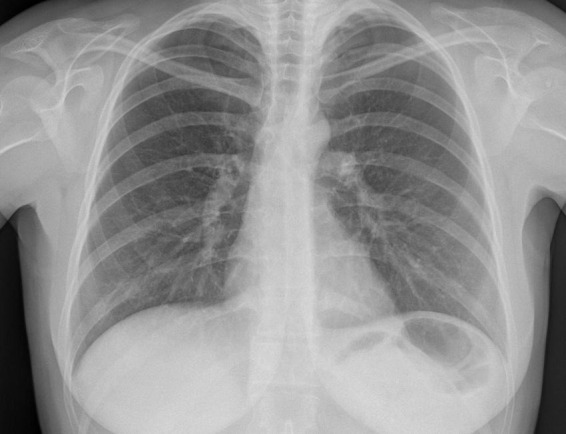
Chest X-ray done at the beginning of IVF treatment.

**FIGURE 2:**
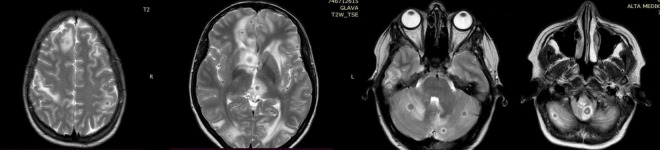
Diffuse nodular changes in the brain parenchyma seen on the nuclear magnetic resonance (NMR).

**FIGURE 3:**
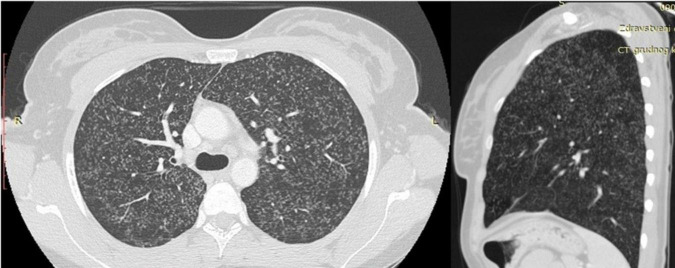
Computerized tomography (CT) of the chest with visible multiple micronodular changes in the lungs.

**FIGURE 4:**
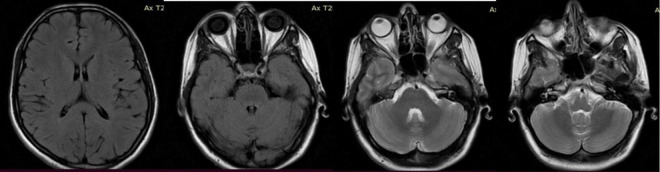
Follow-up brain NMR after 6 months of antituberculosis treatment.

A 28-year-old Caucasian woman, human immunodeficiency virus (HIV) seronegative, vaccinated at birth with the Bacillus Calmette-Guerin (BCG) vaccine, without any previous medical history, became pregnant after the fourth IVF attempt. According to the available clinical data, the patient did not have any multisystemic symptoms during the previous IVF attempts. During her childhood, when she was 7 years old, her father was treated for drug-sensitive tuberculosis (DST), and it is still unknown if she was evaluated at the time as a person from the household. The initial chest X-ray done at the time of the pregnancy initiation was without pathological changes (Figure 1). At the end of the third month of pregnancy, the patient started experiencing dry cough, intermittent high fever up to 39°C, abdominal pain, and vaginal bleeding. The pregnancy was terminated at the end of the first trimester by hysterotomy because of extensive vaginal bleeding, and two stillborn fetuses (male and female, weighing 60 and 80 g) were evacuated from the uterus. Methicillin-resistant *Staphylococcus aureus* (MRSA) was isolated from the uterine cavity. During the following post-partum period, the patient remained febrile despite the treatment with numerous antibiotics regimens containing carbapenems, vancomycin, and piperacillin-tazobactam. One month after delivery she underwent computerized tomography (CT) of pulmonary artery examination, which revealed scattered reticular and micronodular changes in the pulmonary parenchyma without evidence of pulmonary thromboembolic disease. Three months after delivery, the patient started experiencing nausea, vomiting, and weight loss, and in the next 6 months, she started having strong headaches. The nuclear magnetic resonance (NMR) of the brain was performed and showed multiple nodular lesions in the brain parenchyma which were characterized as possible metastatic changes (Figure 2). The patient was finally referred to the tertiary-level hospital institution for further diagnostic evaluation under the suspicion of having a disseminated malignant disease. At the time of hospital admission, the chest X-ray revealed diffuse miliary changes. The CT examination of the chest and upper abdomen revealed diffuse micronodular changes in both lungs (Figure 3). The patient underwent a bronchoscopy examination which showed signs of mild bronchial inflammation. Sputum and tracheobronchial samples were sent for GeneX-pert MTB/RIF assay analysis and came back positive. The antituberculosis treatment was initiated with the standard antituberculosis drug regimen consisting of isoniazid, rifampin, pyrazinamide, and ethambutol. To determine the extent of the disease, positron emission tomography (PET) was performed and revealed the enhanced metabolic activity in the brain, lungs, mediastinum, liver, ileocecal, and genitourinary area. The final microbiologic confirmation of tuberculosis infection we got later was in the form of positive Löwenstein-Jenssen cultures of the sputum, tracheobronchial lavage, blood, urine, and menstrual blood. *Mycobacterium tuberculosis* strain was sensitive to all standard antituberculosis drugs. After the initiation of antituberculosis treatment, the patient experienced a favorable clinical outcome, with the complete resolution of all previously described pathological findings, including the complete resolution of the brain lesions, as was shown on the follow-up NMR of the brain (Figure 4).

## Discussion

It was already known that tuberculosis (TB) could have a tremendous impact on pregnancy outcomes and is an important cause of maternal and fetal morbidity and mortality. The disease progresses more rapidly in pregnant women and can lead to miscarriage (2). Furthermore, 15–30% of pregnant women with TB exhibit hematogenous dissemination and the development of miliary tuberculosis (3). It is also important to emphasize the significance of the possible existence of latent tuberculosis infection, since in the absence of appropriate treatment, there is an estimated lifetime risk of 8–10% for the reactivation of the disease, and that risk varies and can be much higher in case of immunosuppression (4).

As in the case of our patient, the clinical symptoms of infection are often nonspecific, most commonly including fever and cough, which often leads to misdiagnosis and delayed treatment. Also, diagnostic methods such as chest X-ray or chest CT are performed more conservatively during pregnancy because of the radiation exposure risk, so the diagnosis of miliary tuberculosis is often further delayed (5).

The use of IVF methods revolutionized modern infertility worldwide with generally good success rates. It is known that genital tuberculosis (GTB), as a form of extrapulmonary TB, can be a major cause of primary infertility among women in countries with high TB burden (6). The use of IVF and embryo transfer (ET) methods enables the bypassing of fallopian tubes damaged by TB, which consequently can lead to the coexistence of pregnancy and genital TB infection (7). There is an increasing number of reports of cases of miliary tuberculosis in patients who have undergone IVF treatment. The retrospective study of Wang et al. has shown that the incidence of miliary tuberculosis was significantly higher among IVF-ET patients than in the group of patients who have conceived naturally (8). Furthermore, Gai et al. also showed in their retrospective study that women with TB infection during IVF achieved pregnancy were more prone to the hematogenous dissemination of the disease (9). A possible explanation underlines the role of immune and endocrine disorders during pregnancy. It has been shown that during pregnancy cell-mediated immunity has been impaired because of a relative bias toward T-helper type 2 (Th2) immunity (10). This may explain increased susceptibility to certain infections or their reactivation during pregnancy, as in the case of tuberculosis infection. Furthermore, the equilibria between Th1 and Th2 cellular responses are crucial for the determination of the outcome of tuberculosis disease (11).

The role of endocrine mechanisms including effects of progesterone and estrogen could be responsible for immunity response. It is known that progestogens can have a role in a dose-dependent effect on the Th1/Th2 response leading to the reduction of T-cell proliferation and the suppression of host immunity, which is important because of the routine progestogen supplementation after IVF (12). In clinical practice, the progestogen supplementation is commonly given until the 8th to 10th gestational week, and as shown in previous retrospective studies a large proportion of patients develop symptoms a few weeks later, which was seen in our patient (13, 14). High estrogen levels can increase *Mycobacterium tuberculosis* proliferation, and it is also proposed that the existence of increased vascular permeability after pregnancy may also lead to the facilitated hematogenous dissemination of the disease (3). Lastly, the possible role of glucocorticoids, which are given to sensitize the ovaries to gonadotropin stimulation during IVF and their immunosuppressive effects, should not be overlooked in the development of the disease (15).

The differential diagnosis of miliary tuberculosis, especially based only on radiological findings, is broad and complex. Distinguishing miliary changes from widespread metastatic cancer can be a diagnostic challenge, as in our patient’s case (16). Therefore, to make an appropriate and timely diagnosis, it is important to consider including radiological findings, the patient’s signs and symptoms, immune status, and family history.

In the present case, the initial diagnosis of MRSA from the uterine cavity delayed the diagnosis of miliary tuberculosis. Concomitant infection due to MRSA and *Mycobacterium tuberculosis* is uncommon and demonstrates that the diagnosis of miliary tuberculosis can be hidden by the existence of other pathogens (17).

The Republic of Serbia is among low TB burden countries, with an average TB incidence of 7.14/100.000 (18). As was shown in the case of our patient, even in a low TB-burden country, with increased rates of immigration, clinicians should be aware of the increased possibility of the development of miliary tuberculosis in pregnant women, especially in cases of IVF. Furthermore, it is also important to consider the possibility of latent TB infection, and the need for appropriate testing and chemoprophylaxis implementation.

Based on the presented case and available literature data, miliary tuberculosis should be taken into consideration as a possible risk for infertility in the presence of nonspecific symptoms. Nevertheless, in order to be able to prove the presence of a definitive connection between infertility and TB, larger studies are needed to be done in the future. Furthermore, appropriate screening methods for latent tuberculosis infection within households are needed, even in a country with a low TB burden to prevent active disease.

## Data Availability

The original contributions presented in this study are included in this article/supplementary material, further inquiries can be directed to the corresponding author.
